# Effect of Flavin-Containing Monooxygenase Genotype, Mouse Strain, and Gender on Trimethylamine *N*-oxide Production, Plasma Cholesterol Concentration, and an Index of Atherosclerosis[Fn FN3]

**DOI:** 10.1124/dmd.117.077636

**Published:** 2018-01

**Authors:** Sunil Veeravalli, Kersti Karu, Flora Scott, Diede Fennema, Ian R. Phillips, Elizabeth A. Shephard

**Affiliations:** Institute of Structural and Molecular Biology (S.V., F.S., D.F., I.R.P., E.A.S.) and Mass Spectrometry Facility, Department of Chemistry (K.K.), University College London, London, United Kingdom; and School of Biological and Chemical Sciences, Queen Mary University of London, London, United Kingdom (I.R.P.)

## Abstract

The objectives of the study were to determine the contribution, in mice, of members of the flavin-containing monooxygenase (FMO) family to the production of trimethylamine (TMA) *N*-oxide (TMAO), a potential proatherogenic molecule, and whether under normal dietary conditions differences in TMAO production were associated with changes in plasma cholesterol concentration or with an index of atherosclerosis (Als). Concentrations of urinary TMA and TMAO and plasma cholesterol were measured in 10-week-old male and female C57BL/6J and CD-1 mice and in mouse lines deficient in various *Fmo* genes (*Fmo1^−/−^*, *2^−/−^*, *4^−/−^*, and *Fmo5^−/−^*). In female mice most TMA *N*-oxygenation was catalyzed by FMO3, but in both genders 11%–12% of TMA was converted to TMAO by FMO1. Gender-, *Fmo* genotype-, and strain-related differences in TMAO production were accompanied by opposite effects on plasma cholesterol concentration. Plasma cholesterol was negatively, but weakly, correlated with TMAO production and urinary TMAO concentration. *Fmo* genotype had no effect on Als. There was no correlation between Als and either TMAO production or urinary TMAO concentration. Our results indicate that under normal dietary conditions TMAO does not increase plasma cholesterol or act as a proatherogenic molecule.

## Introduction

A correlation between plasma trimethylamine (TMA) *N*-oxide (TMAO) concentrations and atherosclerotic plaque size has been reported in atherosclerosis-prone mice fed on diets supplemented with dietary precursors of TMA (Wang et al., 2011; Koeth et al., 2013). TMAO production is the result of a two-step process, requiring interplay between commensal gut bacteria and the host (Fennema et al., 2016). This process involves the liberation of TMA from dietary precursors, such as choline, carnitine, and TMAO itself (Mitchell et al., 2002; Fennema et al., 2016), and the subsequent host-dependent hepatic *N*-oxygenation of TMA to TMAO (Ayesh et al., 1993). In humans, this oxygenation reaction is catalyzed by flavin-containing monooxygenase (FMO) 3 (Dolphin et al., 1997; Lang et al., 1998). Healthy individuals excrete in their urine ∼95% of total TMA (TMA + TMAO) as TMAO and ∼5% as TMA (Al-Waiz et al., 1987b). Individuals homozygous or compound heterozygous for mutations that severely affect FMO3 activity have impaired *N*-oxygenation of TMA and suffer from the inherited metabolic disorder primary trimethylaminuria (Dolphin et al., 1997; Phillips and Shephard, 2015; Shephard et al., 2015).

There are five functional FMOs in humans: FMO1, 2, 3, 4, and 5 (Phillips et al., 1995; Hernandez et al., 2004). Of these, FMO2 is not expressed in the majority of humans (Dolphin et al., 1998; Veeramah et al., 2008). FMO4 is expressed in very low amounts and little is known about the role of this FMO (Dolphin et al., 1996; Zhang and Cashman, 2006). In human liver, at the time of birth there is a switch in the *FMO* genes expressed: *FMO3* is switched on and *FMO1* is switched off (Dolphin et al., 1996; Koukouritaki et al., 2002). The *FMO1* gene continues to be expressed in humans in the kidney (Dolphin et al., 1996) through the use of an alternative promoter (Shephard et al., 2007). Therefore, after birth, humans primarily express two *FMO* genes in their liver, *FMO3* and *FMO5* (Dolphin et al., 1996; Overby et al., 1997; Koukouritaki et al., 2002; Zhang and Cashman, 2006). However, all other mammals studied continue to express *FMO1* in adult liver (Phillips et al., 2007).

In mice, there is an age-related gender difference in the expression of *Fmo* genes in the liver: male mice, post 5–6 weeks of age, switch off the expression of *Fmo3*, and thus represent natural liver-specific knockouts for FMO3 (Falls et al., 1995; Janmohamed et al., 2004). Adult male mice consequently express two *Fmo* genes, *Fmo1* and *Fmo5*, in liver. In contrast, adult female mice, in addition to expressing *Fmo1* and *Fmo5*, continue to express *Fmo3* in liver. The gender-related differences in *Fmo* gene expression in mice are due to the influence of sex steroids (Falls et al., 1997).

Reports associating TMAO with atherosclerosis and increased risk of cardiovascular disease (Wang et al., 2011; Koeth et al., 2013) are largely based on studies involving concentrations of precursors of TMA or of TMAO itself far in excess of those found under normal dietary conditions and it is not clear whether atherosclerosis risk factors are influenced by physiologically relevant concentrations of TMAO.

Here, we report the use of male and female mice of two different strains and knockout mouse lines deficient in various FMOs (*Fmo1^−/−^*, *2^−/−^*, *4^−/−^*, and *Fmo5^−/−^*) to determine the contribution of particular FMOs to the production of TMAO in vivo and to investigate whether, under normal dietary conditions, differences in TMAO production are associated with changes in plasma cholesterol concentration or with an index of atherosclerosis (Als). We found that in females conversion of TMA to TMAO is catalyzed mainly by FMO3, an enzyme that is absent from the liver of adult males (Falls et al., 1995; Janmohamed et al., 2004), but in both genders FMO1 contributes to TMAO production. Differences in TMAO production were accompanied by opposite effects on plasma cholesterol concentration and were not correlated with Als. Our results indicate that at physiologically relevant concentrations TMAO does not act as a proatherogenic molecule in mice.

## Materials and Methods

### 

#### Animal Maintenance.

C57BL/6J wild-type (WT) mice and two knockout mouse lines (*Fmo1^−/−^*, *2^−/−^*, *4^−/−^*, and *Fmo5^−/−^*, each of which was back-crossed for eight generations on the C57BL/6J background) were bred at University College London. Generation of the knockout mouse lines has been described previously (Hernandez et al., 2009; Gonzalez Malagon et al., 2015). CD-1 mice were purchased from Charles River (Margate, Kent, United Kingdom) and allowed to acclimatize for 14 days before sample collection. All mice were housed in the same room and given free access to water and fed ad libitum with a standard chow diet (2018 Teklad Global 18% Protein Rodent Diet, Harlan Laboratories, Inc., Madison, WI), which contained a choline content of 1.2 g/kg. Blood and urine were collected from 10-week-old male and female mice between 10:00 AM and 12:00 PM. Animal procedures were carried out in accordance with the U.K. Animal Scientific Procedures Act (https://www.gov.uk/government/publications/consolidated-version-of-aspa-1986) and with local ethics committee approval (Animal Welfare and Ethical Review Body) (http://www.ucl.ac.uk/research/integrity/animal-research-accordion/awerb-responsibilities) and appropriate Home Office licenses.

#### Urine Analyses.

Concentrations of TMA, TMAO, and creatinine in urine samples were determined by capillary liquid chromatography electrospray ionization mass spectrometry as described previously (Veeravalli et al., 2017). Production of TMAO was assessed by determining the percentage of total TMA excreted as TMAO, using the following formula: [TMAO/(TMA + TMAO)] × 100 (Al-Waiz et al., 1989). To control for differences in urine concentration, urinary TMAO and TMA concentrations were expressed relative to urinary creatinine concentration.

#### Plasma Metabolites.

Blood samples were collected and plasma isolated as described previously (Hough et al., 2002). Concentrations of total cholesterol and high-density lipoprotein (HDL) and low-density lipoprotein (LDL) cholesterol were determined via an autoanalyzer at the Medical Research Council Mammalian Genomics Unit (Harwell, Oxfordshire, United Kingdom) as described previously (Hough et al., 2002). Als was calculated as (total plasma cholesterol − HDL cholesterol)/HDL cholesterol (Gao et al., 2014).

#### Quantitative Real-Time Polymerase Chain Reaction.

RNA was isolated from liver and individual mRNAs were quantified by quantitative real-time polymerase chain reaction, according to the ΔΔCT method (Liu and Saint, 2002), as described previously (Veeravalli et al., 2014). Primers for SREBP-2, 3-hydroxy-3-methylglutaryl-CoA reductase, CYP7A1, CYP27A1, SR-B1, ABCG5, ABCG8, and ABCB11 were as described previously (Gonzalez Malagon et al., 2015). Additional forward and reverse primer sequences for 3-hydroxy-3-methylglutaryl-CoA synthase 1 (HMGCS1) and squalene synthase were the following: HMGCS1 forward 5′GTGGCACCGGATGTCTTTG3′ and HMGCS1 reverse 5′ACTCTGACCAGATACCACGTT3′, respectively; and squalene synthase forward 5′ATGGAGTTCGTCAAGTGTCTAGG3′ and squalene synthase reverse 5′CGTGCCGTATGTCCCCATC3′, respectively. The use of a geNormTM kit and geNorm software (Primer Design Ltd., Southampton, Hampshire, United Kingdom), as described previously, identified glyceraldehyde-3-phosphate dehydrogenase as the most suitable housekeeping gene for liver (Veeravalli et al., 2014).

#### Statistical Analyses.

Values are given as mean ± S.E.M. Statistical significance was determined using an unpaired two-tailed student’s *t* test. Statistical significance is represented as *P < 0.05; **P < 0.01; ***P < 0.001. Correlations were assessed using Pearson’s *r*.

## Results

### 

#### Mouse Gender, *Fmo* Genotype, and TMAO Production.

**Fig. 1.**
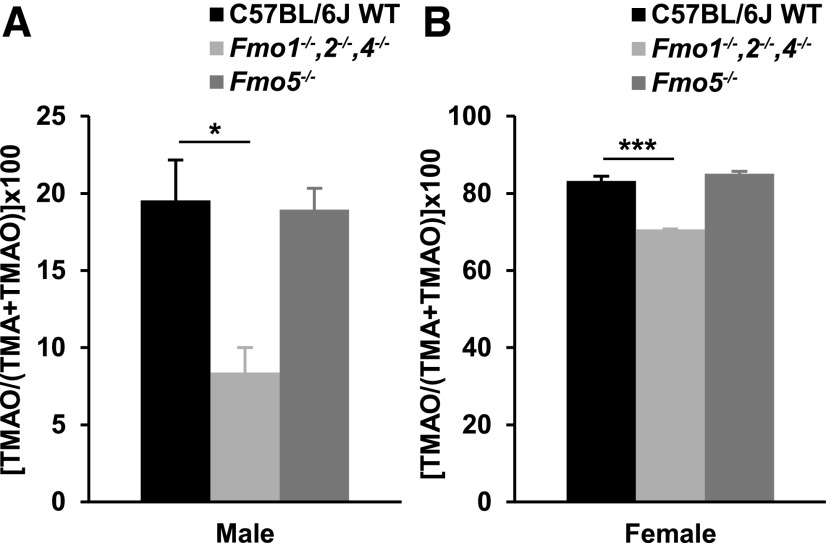
Effect of *Fmo* genotype and gender on TMAO production. Urinary excretion of TMAO as a percentage of total TMA (TMA + TMAO) in male (A) and female (B) mice. *n* = 3–5. *P < 0.05; ***P < 0.001.

Urine from male and female C57BL/6J WT mice and two *Fmo*-knockout lines, *Fmo1^−/−^*, *2^−/−^*, *4^−/−^*, and *Fmo5^−/−^*, was analyzed for TMAO and TMA content. The FMO isoforms expressed in the livers of these animals at 10 weeks of age are given in Table 1. Irrespective of *Fmo* genotype, the percentage of total TMA (TMA + TMAO) excreted as TMAO was far less in male than in female mice (Fig. 1, A and B).

**TABLE 1:** FMO isoforms expressed in the livers of 10-week-old WT and *Fmo*-knockout mouse lines

Mouse Line	Male	Female
C57BL/6J WT	FMO1, FMO5	FMO1, FMO3, FMO5
*Fmo1^−/−^*, *2^−/−^*, *4^−/−^*	FMO5	FMO3, FMO5
*Fmo5^−/−^*	FMO1	FMO1, FMO3

In both male and female *Fmo1^−/−^*, *2^−/−^*, and *4^−/−^* mice the proportion of total TMA excreted as TMAO was less than in gender-matched C57BL/6J WT mice (Fig. 1, A and B). In males the proportion of total TMA excreted as TMAO by *Fmo1^−/−^*, *2^−/−^*, and *4^−/−^* mice (8.4% ± 1.6%) was 56% less than by WT mice (19.5% ± 2.6%), whereas in females TMAO excretion by *Fmo1^−/−^*, *2^−/−^*, and *4^−/−^* mice (70.7% ± 0.1%) was 14% less than by WT mice (83.2% ± 1.3%). There was no increase in expression of the *Fmo3* gene in the liver of *Fmo1^−/−^*, *2^−/−^*, and *4^−/−^* mice, in response to deletion of *Fmo* genes (Hernandez et al., 2009). The results, therefore, indicate that in mice FMO 1, 2, or 4 contributes to the *N*-oxygenation of TMA. It is most likely to be FMO1 because this enzyme is expressed in adult mouse liver, the site of TMA *N*-oxygenation, whereas FMOs 2 and 4 are not expressed in this tissue (Janmohamed et al., 2004). Thus, in both male and female mice, 11%–12% of TMA is converted to TMAO by FMO1. In *Fmo5^−/−^* mice the proportion of total TMA excreted as TMAO was the same as that in WT mice, in both males and females, indicating that FMO5 plays no role in the conversion of TMA to TMAO in vivo.

#### Mouse Gender, *Fmo* Genotype, and Plasma Cholesterol Concentration.

Increased concentration of TMAO has been linked to the formation of proatherogenic plaques (Wang et al., 2011), of which cholesterol is an important component. The gender- and *Fmo* genotype–related differences in TMAO production (Fig. 1) led us to investigate the effect of gender and *Fmo* genotype on plasma cholesterol concentrations. Plasma concentrations of total, HDL, and LDL cholesterol were determined in male and female C57BL/6J WT, *Fmo1^−/−^*, *2^−/−^*, *4^−/−^*, and *Fmo5^−/−^* mice (Table 2). In WT mice and in both knockout mouse lines females had significantly lower plasma concentrations of total and HDL cholesterol than did their male counterparts, but the plasma concentration of LDL cholesterol was similar in male and female mice. Therefore, the greater production of TMAO in female mice was accompanied by significantly lower plasma concentrations of total and HDL cholesterol.

**TABLE 2:** Effect of *Fmo* genotype and gender on plasma concentrations of total, HDL, and LDL cholesterol The *P* values are for comparisons of the values for each mouse line or strain vs. those for C57BL/6J WT mice of the same gender: *n* = 3–5; *P < 0.05; **P < 0.01; ***P < 0.001.

Mouse Line/Strain	Gender	Cholesterol Concentration		
		Total	HDL	LDL
		*mmol/l*	*mmol/l*	*mmol/l*
C57BL/6J WT	Male	2.88 ± 0.09	2.09 ± 0.07	0.54 ± 0.02
C57BL/6J WT	Female	2.41 ± 0.13	1.67 ± 0.08	0.48 ± 0.03
*Fmo1^−/−^*, *2^−/−^*, *4^−/−^*	Male	4.03 ± 0.12***	3.05 ± 0.09***	0.61 ± 0.03
*Fmo1^−/−^*, *2^−/−^*, *4^−/−^*	Female	3.41 ± 0.13**	2.39 ± 0.11**	0.69 ± 0.01**
*Fmo5^−/−^*	Male	2.63 ± 0.05*	1.88 ± 0.03*	0.52 ± 0.01
*Fmo5^−/−^*	Female	2.21 ± 0.11	1.53 ± 0.08	0.48 ± 0.03
CD-1	Male	3.76 ± 0.26*	2.28 ± 0.21	0.66 ± 0.05
CD-1	Female	3.21 ± 0.17*	2.14 ± 0.15*	0.67 ± 0.05*

Both male and female *Fmo1^−/−^*, *2^−/−^*, and *4^−/−^* mice had higher plasma concentrations of total and HDL cholesterol than did their WT counterparts, and in females the plasma concentration of LDL cholesterol was also higher than in WT mice (Table 2). Based on the tissue-specific expression patterns of FMOs in mice (Janmohamed et al., 2004), the higher plasma cholesterol concentration observed in *Fmo1^−/−^*, *2^−/−^*, and *4^−/−^* mice is likely to be due to disruption of the *Fmo1* gene, which is expressed in liver, and not to disruption of *Fmo2* and *Fmo4*, which are not expressed in liver. Consequently, our results indicate that in mice FMO1 plays a role in the regulation of plasma cholesterol concentration. Comparison of *Fmo5^−/−^* and WT mice revealed that plasma concentrations of total and HDL cholesterol were similar in females and lower in males (Table 2), indicating that in males there is a role for FMO5 in promoting plasma cholesterol concentration.

#### Mouse Gender, *Fmo* Genotype, and Atherosclerosis Index.

**Fig. 2.**
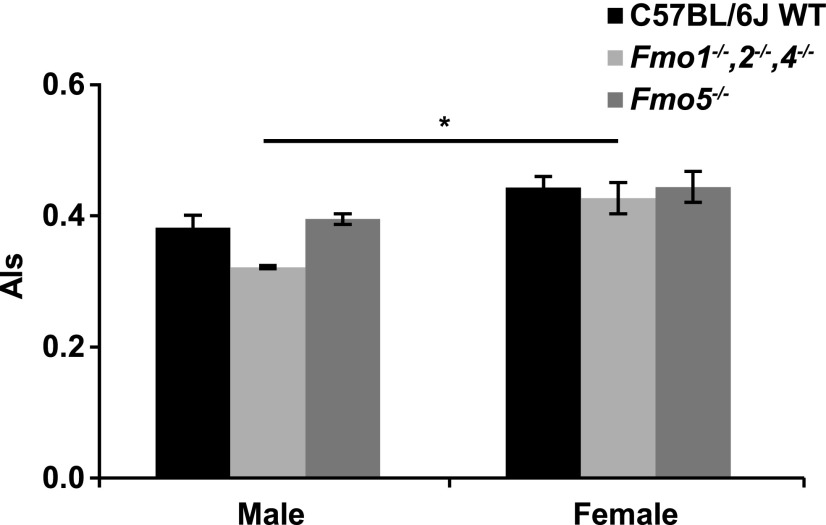
Effect of *Fmo* genotype, gender, and mouse strain on Als. *n* = 3–5. *P < 0.05.

We next investigated the effect of *Fmo* genotype on Als. There was no significant difference in Als between C57BL/6J WT mice and either of the two knockout lines, in either male or female animals (Fig. 2). Similarly, there was no difference in Als between males and females of either WT or *Fmo5^−/−^* mice, despite production of TMAO being 4-fold greater in females (Fig. 1). However, in *Fmo1^−/−^*, *2^−/−^*, and *4^−/−^* mice, Als was ∼26% lower in males than in females (Fig. 2).

#### Mouse Strain, TMAO, Plasma Cholesterol, and Als.

We also investigated the relationship between TMAO production, plasma total cholesterol, and Als in another mouse strain, CD-1. In males, the urinary concentration of TMAO (mM/mM creatinine) was 0.18 ± 0.02 (*n* = 5) for CD-1 mice and 0.70 ± 0.03 (*n* = 4) for C57BL/6J WT mice. For females the values were 0.66 ± 0.06 (CD-1, *n* = 6) and 2.73 ± 0.20 (C57BL/6J WT, *n* = 4). Therefore, for both genders the urinary concentration of TMAO of CD-1 mice was ∼75% less than that of C57BL/6J WT mice (*P* < 0.0001), and as was the case for C57BL/6J WT mice it was higher in female than in male animals (*P* < 0.0001).

The plasma concentration of total cholesterol of CD-1 mice was significantly higher than that of C57BL/6J WT mice in both genders (Table 2). In males, Als was significantly higher in CD-1 (0.68 ± 0.08, *n* = 5) than in C57BL/6J WT (0.38 ± 0.02, *n* = 4) (*P* < 0.05), but not in females (CD-1: 0.51 ± 0.04, *n* = 6; C57BL/6J WT: 0.44 ± 0.02, *n* = 4). Therefore, although CD-1 mice had substantially lower urinary concentrations of TMAO than did C57BL/6J mice, they had higher plasma concentrations of total cholesterol, in both genders, and in males they had had higher Als.

#### Correlations of TMAO with Plasma Cholesterol or Als.

Our results indicate an inverse relationship between TMAO production and the plasma concentration of total cholesterol. Therefore, we investigated whether there was a correlation between total plasma cholesterol concentration and either TMAO production, which is measured by the percentage of total TMA excreted as TMAO, or the urinary concentration of TMAO. Analysis of male and female C57BL/6J WT, CD-1 WT, *Fmo1^−/−^*, *2^−/−^*, *4^−/−^*, and *Fmo5^−/−^* mice (a total of 34 animals) showed a weak negative correlation between plasma total cholesterol concentration and both TMAO production and urinary TMAO concentration (Supplemental Fig. 1, A and B; Table 3). There was no correlation between Als and either TMAO production or urinary TMAO concentration (Supplemental Fig. 1, C and D; Table 3).

**TABLE 3:** Correlation between plasma total cholesterol concentration or Als and TMAO Each correlation analysis was performed on a total of 34 animals. Results from individual animals are shown in Supplemental Fig. 1.

Correlation	*r*	*R*^2^	*P* value
Plasma total cholesterol vs. [TMAO/(TMA + TMAO)] × 100	−0.357	0.127	0.038
Plasma total cholesterol vs. TMAO/creatinine	−0.464	0.215	0.006
Als vs. [TMAO/(TMA + TMAO)] × 100	0.136	0.019	0.442
Als vs. TMAO/creatinine	−0.151	0.023	0.395

#### Quantification of mRNAs Encoding Proteins Involved in Cholesterol Synthesis, Uptake, Transport, or Disposition.

*Fmo1^−/−^*, *2^−/−^*, and *4^−/−^* mice, both male and female, had higher plasma concentrations of total and HDL cholesterol compared with their WT and *Fmo5^−/−^* counterparts (Table 2). To attempt to identify a potential basis for the elevated plasma concentrations of cholesterol and HDL cholesterol in *Fmo1^−/−^*, *2^−/−^*, and *4^−/−^* mice, mRNAs encoding proteins involved in cholesterol biosynthesis, cholesterol uptake and transport, and bile acid synthesis and secretion were quantified by quantitative real-time polymerase chain reaction in livers of male *Fmo1^−/−^*, *2^−/−^*, *4^−/−^*, and C57BL/6J WT mice (Table 4).

**TABLE 4:** Relative difference in abundance of mRNAs in liver of C57BL/6J WT and *Fmo1^−/−^*, *2^−/−^*, and *4^−/−^* mice

mRNA	Relative expression, Knockout vs. WT
Cholesterol biosynthesis	
HMG CoA synthase	0.92 ± 0.22
HMG CoA reductase	0.94 ± 0.25
Squalene synthase	1.01 ± 0.12
SREBP-2	2.41 ± 0.36*
Cholesterol uptake and transport	
SR-B1	2.53 ± 0.36**
ABCG5	1.06 ± 0.35
ABCG8	1.24 ± 0.26
Bile acid synthesis	
CYP7A1	3.12 ± 1.91
CYP27A1	1.67 ± 0.79
Canalicular lipid transport	
ABCB11	4.40 ± 0.95*

*P < 0.05; **P < 0.01.

The mRNA for SREBP-2, a transcription factor that upregulates expression of genes involved in cholesterol synthesis, was more abundant in *Fmo1^−/−^*, *2^−/−^*, and *4^−/−^* than in C57BL/6J WT animals (Table 4). Although relatively small, the increased abundance of the mRNA for this transcription factor may contribute to the elevated plasma cholesterol observed in *Fmo1^−/−^*, *2^−/−^*, and *4^−/−^* mice. However, the abundance of mRNAs encoding proteins involved in cholesterol biosynthesis was not increased (Table 4).

SR-B1 mRNA was more abundant in the *Fmo1^−/−^*, *2^−/−^*, and *4^−/−^* mice (Table 4). Because of the role of SR-B1 in promoting cholesterol uptake (Varban et al., 1998; Ji et al., 1999) the increase in the abundance of its mRNA may be an attempt to reduce the elevated concentration of plasma HDL cholesterol in *Fmo1^−/−^*, *2^−/−^*, and *4^−/−^* mice. The mRNA for ABCB-11, which encodes the bile salt export pump located on the canalicular membrane of hepatocytes, a protein that controls the rate-limiting step in hepatic bile secretion (Henkel et al., 2013), was more abundant in the *Fmo1^−/−^*, *2^−/−^*, and *4^−/−^* mice (Table 4). The increase in ABCB-11 mRNA may be in response to increased SR-B1-mediated cholesterol uptake and suggests that bile flow from the liver to the intestine may be greater in *Fmo1^−/−^*, *2^−/−^*, and *4^−/−^* mice than in WT mice. However, there was no difference between *Fmo1^−/−^*, *2^−/−^*, *4^−/−^*, and WT mice in the abundance of mRNAs encoding either CYP7A1 or CYP27A1 (Table 4), two enzymes important in bile acid synthesis (Norlin and Wikvall, 2007).

## Discussion

Our results show that female mice produced far more TMAO than did male animals. This marked gender difference is consistent with previous findings (Li et al., 2013) and can be explained by the fact that at 5–6 weeks of age the expression of the gene encoding FMO3, the major enzyme involved in conversion of TMA to TMAO in both human and mouse (Dolphin et al., 1997; Lang et al., 1998; Zhang et al., 2007), is switched off in the liver of male, but not female, mice (Falls et al., 1995; Janmohamed et al., 2004).

In both male and female C57BL/6J WT mice FMO1, but not FMO5, contributed to TMAO production, with 11%–12% of TMA being converted to TMAO by the action of FMO1. Consequently, in females the majority of TMAO was produced by hepatic FMO3, but in males, which lack hepatic FMO3, most of the much lower production of TMAO was derived from the action of FMO1. Our results in vivo are consistent with those found in vitro (Bennett et al., 2013).

Results from *Fmo1^−/−^*, *2^−/−^*, and *4^−/−^* mice indicate that despite the disruption of genes encoding FMO1, FMO2, and FMO4 and the lack of hepatic FMO3 males still produced a small amount of TMAO. The reason for this is unclear, but could be due to the action of FMO3 in tissues other than liver, for instance, adult males continue to express FMO3 in Clara cells of the lung (Janmohamed et al., 2004). Other possibilities include the action of non-FMO enzymes or the production of TMAO from TMA by gut bacteria (reviewed by Fennema et al., 2016).

In contrast to mice and other mammals investigated, humans do not express FMO1 in liver after birth (Dolphin et al., 1996; Koukouritaki et al., 2002). Thus, in adult humans FMO1 cannot contribute to hepatic TMAO production. Although FMO1 is expressed in human kidney (Dolphin et al., 1996) and small intestine (Yeung et al., 2000), conversion of TMA to TMAO was not detected in microsomes isolated from these tissues (Lang et al., 1998). Recombinant human FMO1 can catalyze the conversion of TMA to TMAO but only at high (5 mM) concentrations of TMA (Lang et al., 1998). In humans, the concentration of TMA never reaches the mM range, even in individuals with severe trimethylaminuria (Al-Waiz et al., 1987a); this is in contrast to mice, which excrete TMA in the mM range (Li et al., 2013). Consequently, FMO1 is unlikely to contribute to TMAO production in humans.

Gender-, *Fmo* genotype–, and strain-related differences in TMAO production were in all cases accompanied by opposite effects on plasma total cholesterol concentration. Plasma total cholesterol concentration was negatively, but weakly, correlated with both TMAO production and urinary TMAO concentration.

Our results indicate that both FMO1 and FMO5 are involved in the regulation of plasma cholesterol concentration, but with opposing effects: FMO1 acts to decrease plasma cholesterol concentration in both male and female animals, whereas FMO5 promotes an increase in plasma cholesterol concentration in males. The latter is consistent with our previous finding that FMO5 promotes an age-related increase in plasma cholesterol concentration, which by 30 weeks of age is evident in both genders (Gonzalez Malagon et al., 2015).

Als was not influenced by *Fmo* genotype and, despite relatively large gender-related differences in production of TMAO, in only one of the three mouse lines, *Fmo1^−/−^*, *2^−/−^*, and *4^−/−^*, was Als influenced by gender, and then to a relatively small extent. There was no correlation between Als and either TMAO production or urinary TMAO concentration.

Several studies of mice have implicated TMAO as a proatherogenic molecule. Most of these have been done on mice carrying an *ApoB* transgene or on *ApoE^−/−^* knockout animals (Wang et al., 2011; Bennett et al., 2013; Koeth et al., 2013; Yang et al., 2014) in an attempt to better mimic the situation in humans, a species in which plasma LDL cholesterol constitutes a higher proportion of total cholesterol than is found in mice. Many of these studies involved feeding the animals with precursors of TMA or with TMAO itself in amounts far in excess of normal dietary levels; for instance, in some cases diets were supplemented with concentrations of choline as high as 13 g/kg, more than 10 times that present in the standard chow diet used in our study. In contrast, a study by Mayr et al. (2005) found no significant difference in TMAO concentration in the aortas of 18-month-old *ApoE^−/−^* and *ApoE^+/+^*mice, despite the former having severe aortic atherosclerotic lesions. The lesion formation in *ApoE^−/−^* mice was ascribed not to increased TMAO but to an increase in oxidative stress (Mayr et al., 2005).

A role for TMAO in predisposition to cardiovascular disease is counterintuitive given that the richest dietary source of TMAO is marine fish, the consumption of which has been shown to exert beneficial effects on the circulatory system (Zhang et al., 1999; Takata et al., 2013). Consistent with this, a study of C57BL/6J WT mice found that the increase in Als associated with a high-fat diet was prevented by dietary supplementation with TMAO, suggesting a protective effect of TMAO with regard to atherosclerosis (Gao et al., 2014).

In our study we used C57BL/6J and CD-1 mice and two *Fmo* gene knockout mouse lines (*Fmo1^−/−^*, *2^−/−^*, *4^−/−^*, and *Fmo5^−/−^*) generated on a C57BL/6J background to investigate, under normal dietary conditions, the effect of mouse gender, mouse strain, and *Fmo* genotype on TMAO production, plasma cholesterol concentration, and Als. We elected not to complicate the experimental design or data interpretation by the use of an additional genotype change, such as the disruption of *ApoE* or the presence of an *ApoB* transgene, or by supplementation of diet with TMA precursors. Our results indicate an inverse relationship between TMAO production and the plasma concentration of total cholesterol, and that neither plasma cholesterol concentration nor Als is positively correlated with either TMAO production or urinary TMAO concentration. Indeed, there is a negative, albeit it weak, correlation between plasma cholesterol concentration and both TMAO production and urinary TMAO concentration. Our results, therefore, indicate that under normal dietary conditions TMAO does not act as a proatherogenic molecule.

## Abbreviations

Als: index of atherosclerosis
FMO: flavin-containing monooxygenase
HDL: high-density lipoprotein
HMGCS1: 3-hydroxy-3-methylglutaryl-CoA synthase 1
LDL: low-density lipoprotein
TMA: trimethylamine
TMAO: trimethylamine *N*-oxide
WT: wild type
